# Supplementary material for: Cardiomyocyte-Targeting Peptide to Deliver Amiodarone
Source: Pharmaceutics. 2023 Aug 9;15(8):2107. doi: 10.3390/pharmaceutics15082107 (PMC10459552; doi:10.3390/pharmaceutics15082107)
Supplement: Supplementary file 1 [file pharmaceutics-15-02107-s001.zip › pharmaceutics-2543326-supplementary.pdf]

Cardiomyocyte-Targeting Peptide to Deliver Amiodarone

Maliha Zahid [1,*], Beth Weber [2], Ray Yurko [3], Kazi Islam [3], Vaishavi Agrawal [4], Jack Lopuszynski [1], Hisato Yagi [5] and Guy Salama [2]

Supplemental Materials

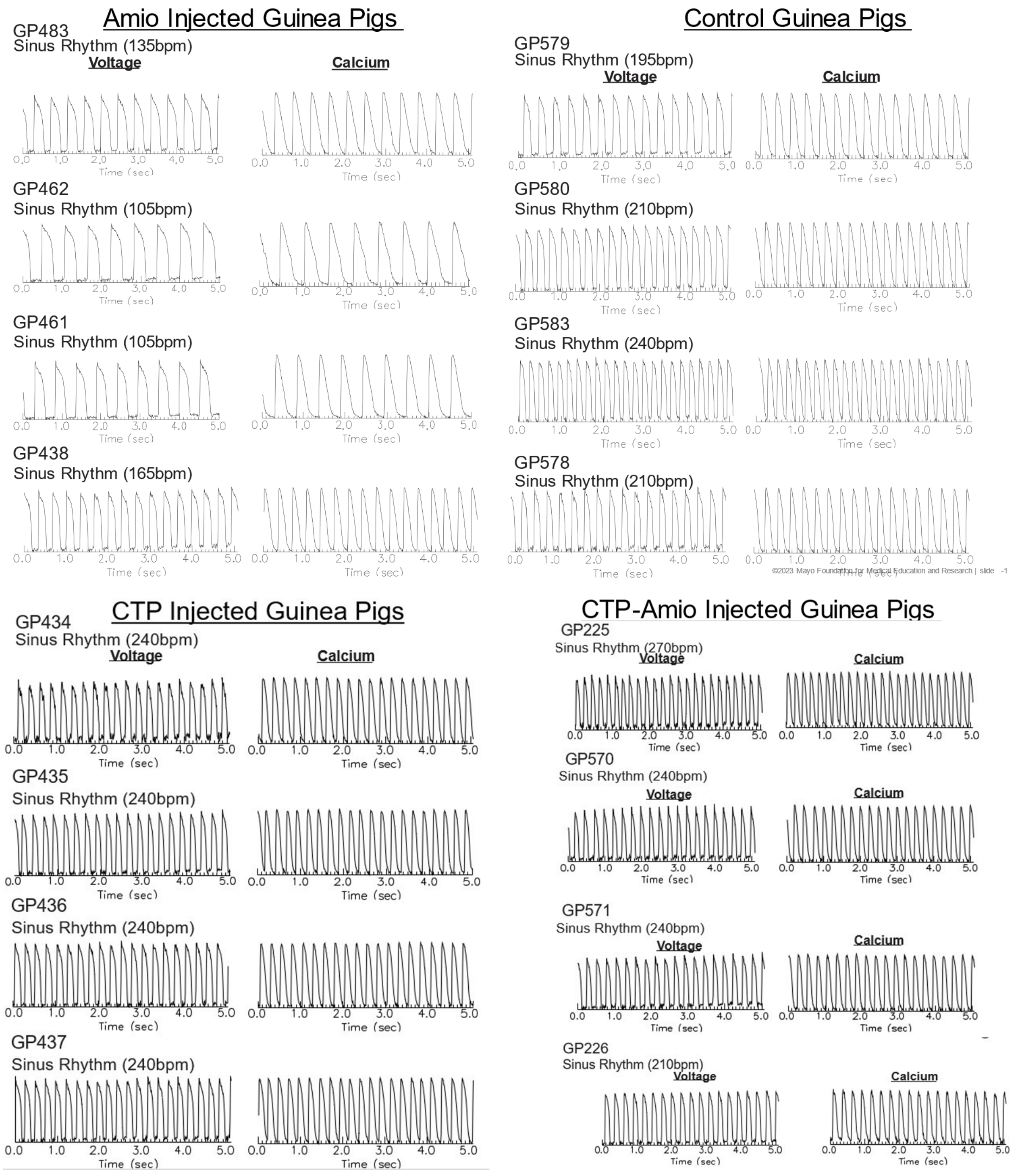

Supplemental Figure S1: Calcium and voltage measurements in each guinea pig injected with amiodarone, control (no injections), CTP, or CTP–amiodarone conjugate.

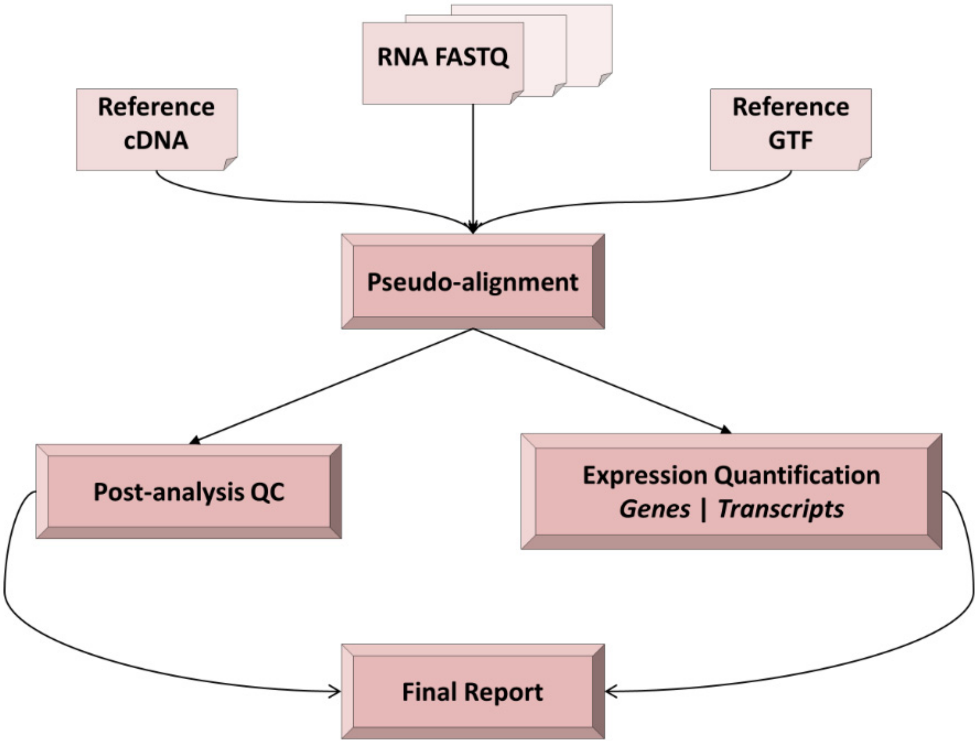

Supplemental Figure S2: Workflow of the RNA sequencing results' analyses.

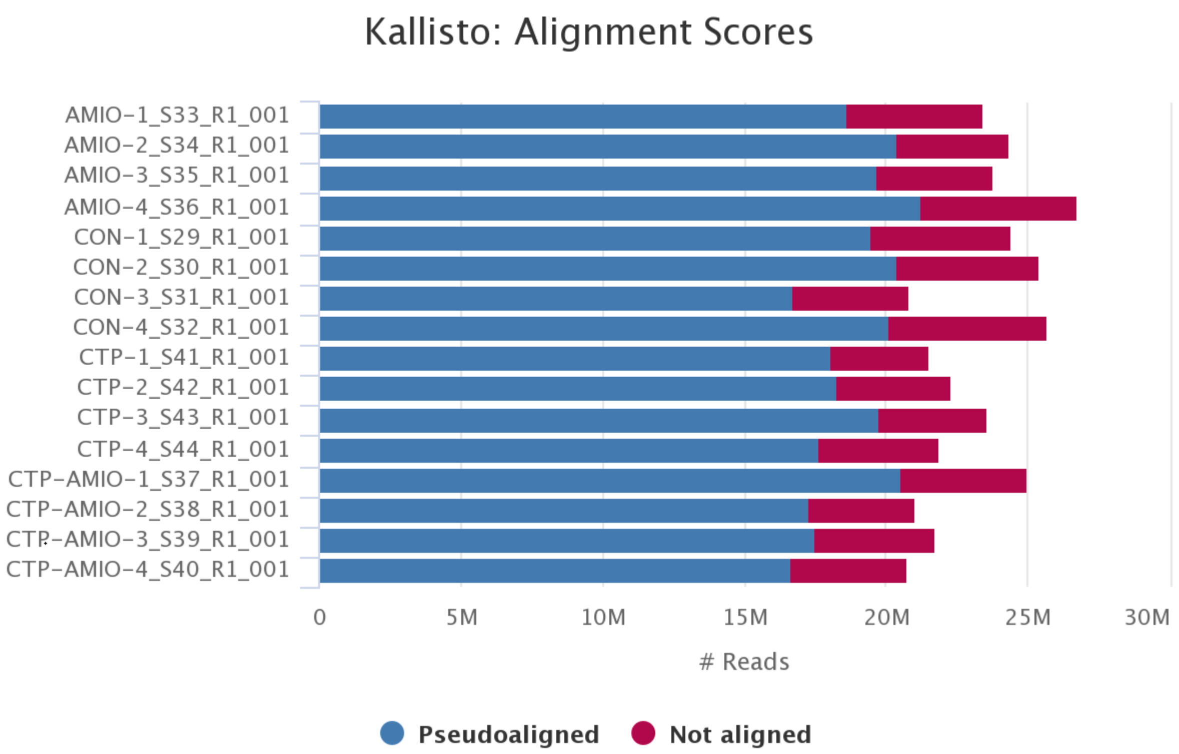

Sample Name	Frag Length	% Aligned	M Aligned
AMIO-1_S33_R1_001	161.3bp	79.5%	18.6
AMIO-2_S34_R1_001	159.2bp	83.6%	20.4
AMIO-3_S35_R1_001	164.7bp	82.9%	19.7
AMIO-4_S36_R1_001	157.2bp	79.5%	21.2
CON-1_S29_R1_001	153.7bp	79.9%	19.5
CON-2_S30_R1_001	155.6bp	80.3%	20.4
CON-3_S31_R1_001	168.1bp	80.3%	16.7
CON-4_S32_R1_001	152.0bp	78.3%	20.1
CTP-1_S41_R1_001	153.3bp	84.0%	18.1
CTP-2_S42_R1_001	156.5bp	81.8%	18.3
CTP-3_S43_R1_001	155.2bp	83.9%	19.8
CTP-4_S44_R1_001	161.9bp	80.6%	17.6
CTP-AMIO-1_S37_R1_001	157.4bp	82.1%	20.5
CTP-AMIO-2_S38_R1_001	153.9bp	82.1%	17.3
CTP-AMIO-3_S39_R1_001	163.7bp	80.7%	17.5
CTP-AMIO-4_S40_R1_001	158.0bp	80.3%	16.7

Supplemental Figure S3: Kalisto alignment of the samples and the resulting scores for each sample.

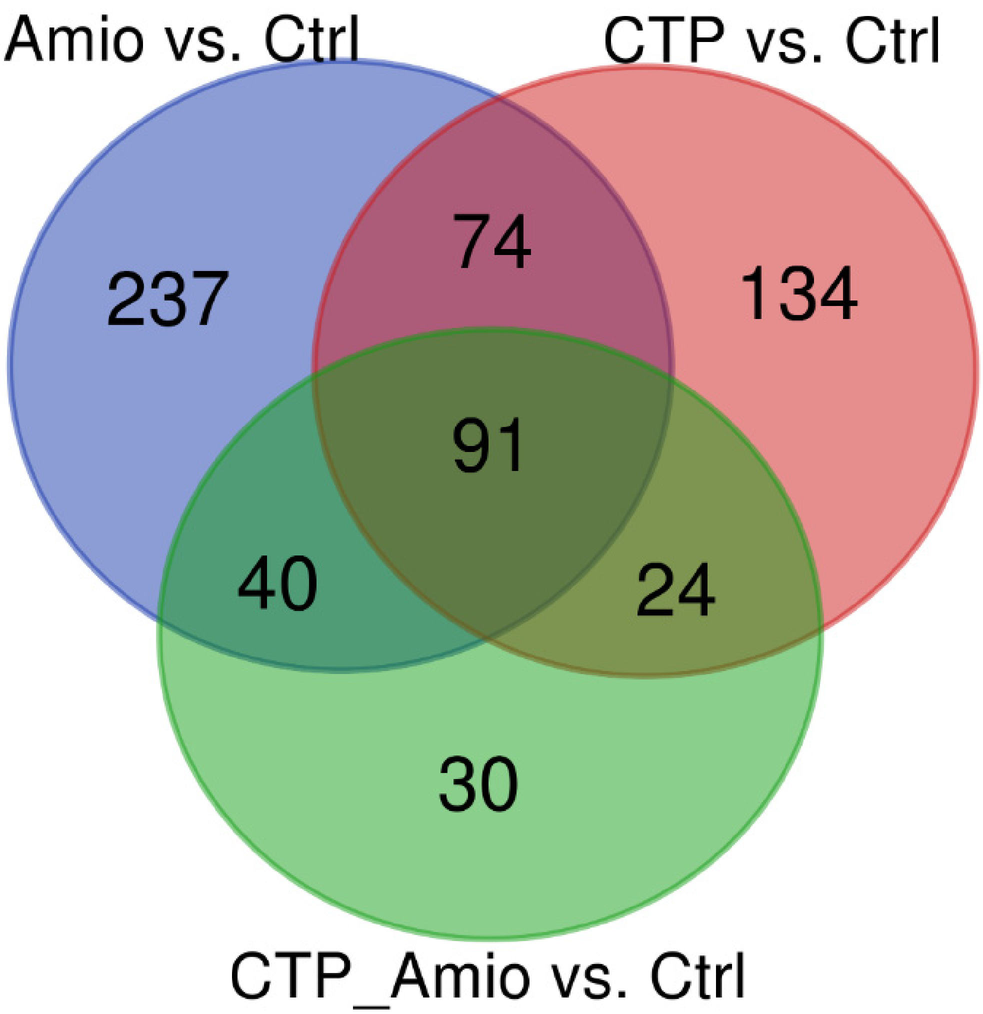

Supplemental Figure S4: Common and unique differentially expressed genes between amiodarone, CTP, CTP–amio and the control guinea pig hearts. The Venn diagram shows the overlap between the three differentially expressed gene sets.

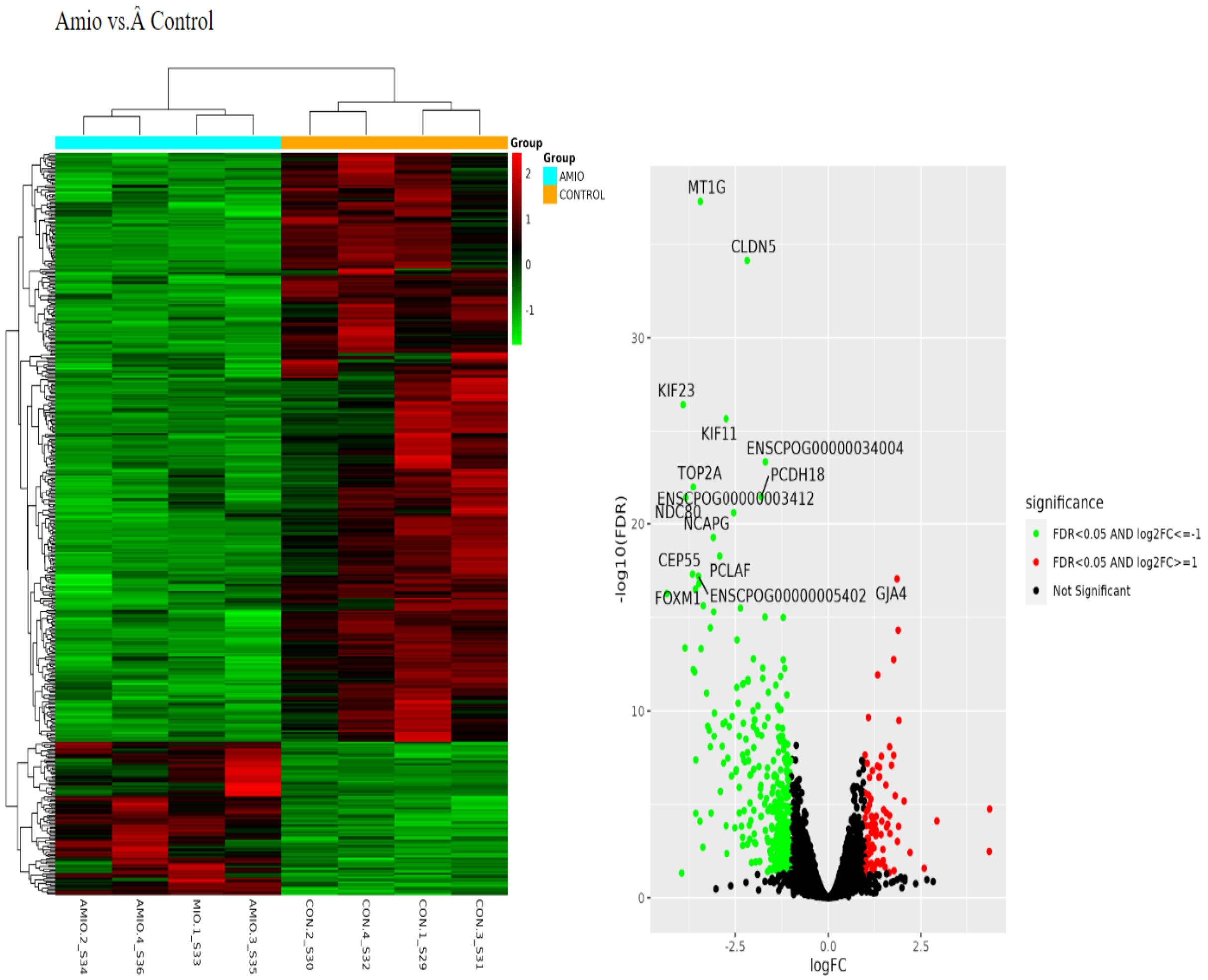

<table><tr><td>Supplemental Figure S5: Heatmap of the differentially expressed genes in guinea pig hearts of amiodarone-treated versus control animals.</td></tr></table>

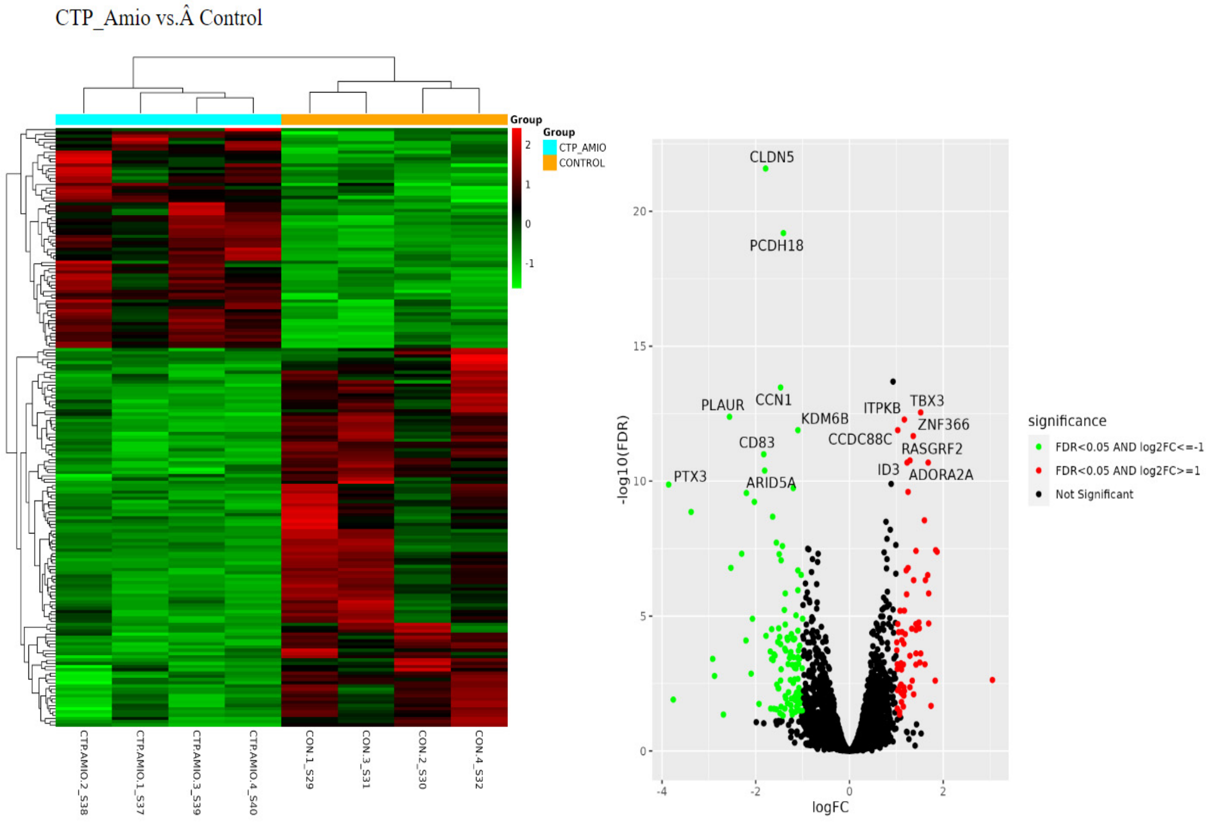

Supplemental Figure S6: Heatmap of differentially expressed genes in guinea pig hearts of CTP–amio-treated versus control animals.

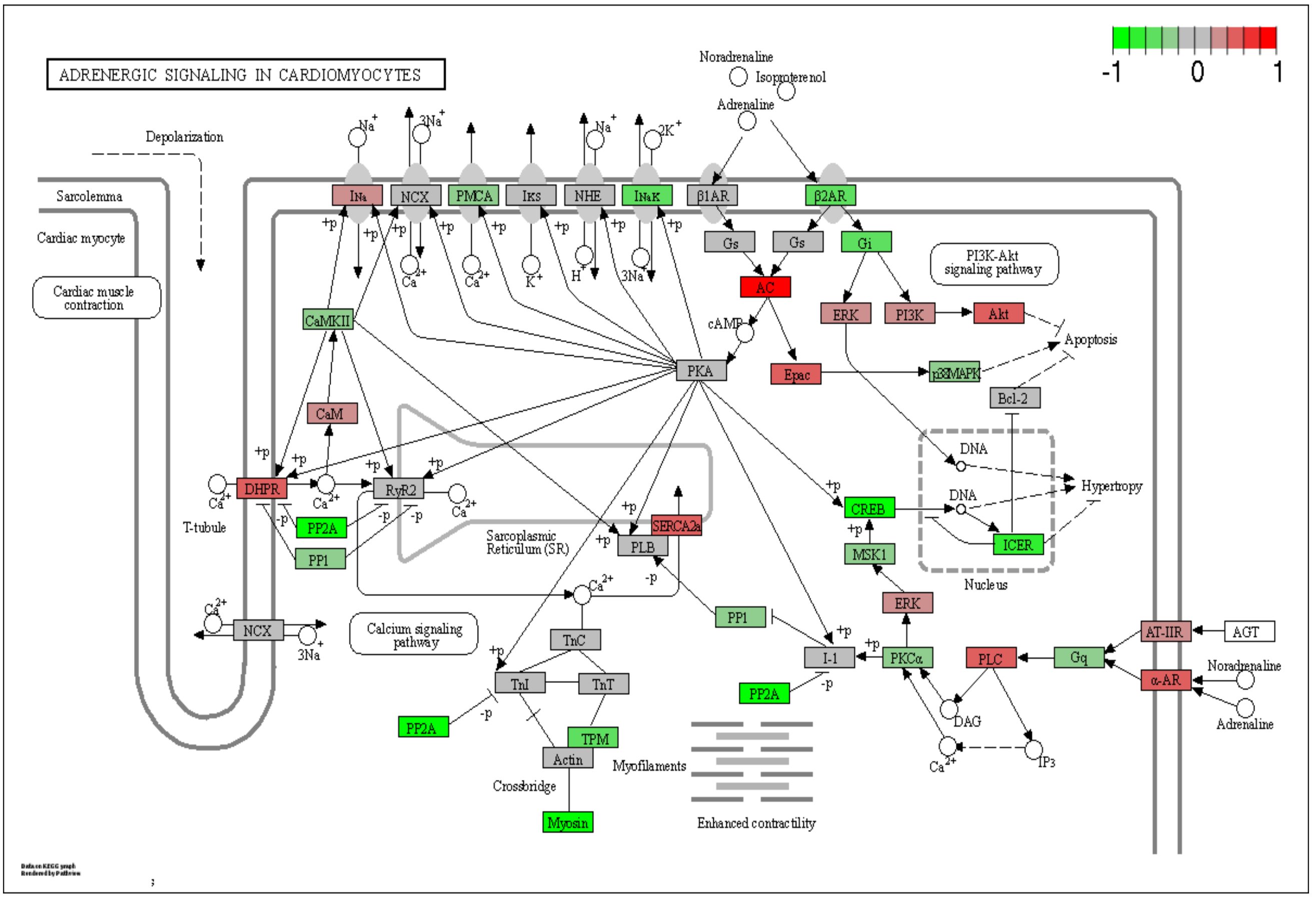

Supplemental Figure S7: Differentially expressed genes in CTP–amio-treated guinea pig hearts compared with control hearts. The beta 2-adrenergic receptor is significantly downregulated, whereas the alpha-adrenergic receptor and calcium-handling channel genes (*DHPR* and *SERCA2a*) are significantly upregulated (all circled in black).
